# Unraveling the Mechanism of High N_2_ Selectivity in Ammonia Selective Catalytic Oxidation on Pt-V Tandem Catalyst

**DOI:** 10.3390/ma18081782

**Published:** 2025-04-14

**Authors:** Yu Gao, Pingshang Li, Wan Mei

**Affiliations:** 1 China Waterborne Transport Research Institute, Beijing 100083, China; 2 School of Environment, Beijing Jiaotong University, Beijing 100044, China; bjulps@163.com (P.L.); meiw177@126.com (W.M.)

**Keywords:** Pt-V tandem catalyst, selective catalytic oxidation of NH_3_, N_2_ selectivity, V loading amount

## Abstract

V_0.5_/Pt/TiO_2_ tandem catalysts exhibit both an outstanding low-temperature NH_3_ conversion rate and high N_2_ selectivity in NH_3_-SCO reactions, but the mechanism of high N_2_ selectivity remains unclear. In this work, V*_x_*/Pt/TiO_2_ tandem catalysts were synthesized through a two-step impregnation–deposition method. The modulating effect of the V loading mount on NH_3_-SCO performance was evaluated, and the relevant reaction mechanism was explored systematically. The results demonstrated that the synergistic effect of tandem NH_3_ oxidation and NH_3_-SCR reactions could be regulated by changing the V loading amount, thereby modulating N_2_ selectivity. Compared with other V*_x_*/Pt/TiO_2_ catalysts and previously reported SCO catalysts, the V_0.5_/Pt/TiO_2_ catalyst with a V loading amount of 0.5 wt.% exhibited outstanding NH_3_-SCO performance, which achieved complete NH_3_ conversion and >90% of N_2_ selectivity within a range of 250–450 °C. XPS, NH_3_-TPD, and O_2_-TPD results suggested that the increase in the V loading amount from 0.1 wt.% to 0.5 wt.% was conducive to increasing the relative contents of Pt^0^ and V^5+^ species, as well as the amount of acid sites, oxygen species, and oxygen vacancies. Consequently, the synergistic effect of tandem NH_3_ oxidation and NH_3_-SCR reactions was significantly enhanced, enabling the catalyst to exhibit excellent N_2_ selectivity. A further increase in the V loading amount from 0.5 wt.% to 0.9 wt.% would bring about the opposite effect to the above, resulting in a decline in catalytic performance. In situ DRIFTS results showed that a V loading amount of 0.5 wt.% was beneficial for -NH_2_ species to participate in NH_3_-SCO reactions, thereby boosting N_2_ selectivity.

## 1. Introduction

In response to increasingly stringent CO_2_ emission reduction regulations, the international shipping industry is exploring the application of NH_3_-fuel engines as the main engines for large merchant ships [1,2,3]. However, the serious environmental risks caused by NH_3_ emissions from NH_3_-fuel engines have become one of the main obstacles limiting the application of NH_3_-fuel engines on board [3,4,5,6,7,8]. It is urgent to explore practical and efficient technologies for removing NH_3_ from high-temperature flue gas to meet the needs of NH_3_-powered ships. At present, the selective catalytic oxidation (SCO) technique is considered more promising than other techniques in meeting the above needs [9,10,11]. This is because SCO technology can effectively convert NH_3_ into N_2_ through Equation (1) at relatively high temperatures and space velocities.

(1) $$4NH_{3}+{3O}_{2}\to {2N}_{2}+{6H}_{2}O\;$$

The NH_3_ conversion performance of SCO technology mainly depends on the design of the catalyst. During the past several decades, numerous types of NH_3_-SCO catalysts have been reported, which can be mainly classified into noble metal catalysts and transition metal oxide catalysts. Noble metal catalysts exhibit an outstanding NH_3_ conversion performance at low temperatures, which is attributed to their strong catalytic oxidation activity for NH_3_ [11,12,13,14,15]. But noble metal catalysts are prone to excessively oxidizing NH_3_ into NO*_x_*, resulting in a low N_2_ selectivity [13,14,15]. Transition metal oxide catalysts exhibit high N_2_ selectivity due to their moderate catalytic oxidation activity for NH_3_, but this makes it difficult to achieve a good NH_3_ conversion performance at low temperatures [15,16,17].

It is considered that the mainstream approach to designing SCO catalysts with excellent performance is to simultaneously pursue good NH_3_ conversion performance and high N_2_ selectivity over a broad temperature range. But this remains difficult to achieve at present. Aiming to synthesize SCO catalysts with excellent performance, the combination of transition metal oxides with a small amount of noble metal components was proposed as a strategy [18,19,20,21,22,23]. Its operation relies on an internal selective catalytic reduction (i-SCR) mechanism, which is mainly composed of the following two tandem reactions: Under catalysis of noble metal components, NH_3_ is oxidized to NO by O_2_ (Equation (2)). Subsequently, NO reacts with NH_3_ under the action of transition metal oxide components through SCR reaction (Equation (3)).

(2) $$4NH_{3}+5O_{2}\to 4\mathrm{NO}+6H_{2}O$$

(3) $$4\mathrm{NO}+4NH_{3}+O_{2}\to {4N}_{2}+{6H}_{2}O$$

For the design of catalysts employing the above strategy, the selection of suitable noble metal and transition metal components is deemed to be of great importance. In recent years, Pt-V tandem catalysts have received extensive attention due to their high potential for achieving good NH_3_ conversion performance and high N_2_ selectivity simultaneously. Byun et al. introduced 0.3 wt.% Pt into the V_3_W_2_/TiO_2_ catalyst. The results showed that, within the temperature range of 330–380 °C, the Pt_0.3_V_3_W_2_/TiO_2_ catalyst could achieve complete conversion of NH_3_ and the corresponding N_2_ selectivity was higher than 90.0% [20]. The main reason for this was the synergistic effect between NH_3_-SCO and CO-SCR reactions. Kim et al. reported that the addition of 2 wt.% V into the Pt_0.1_/TiO_2_ catalyst could enhance the NH_3_-SCO performance. The Pt_0.1_/V_2_/TiO_2_ catalyst derived through optimization completely converted NH_3_ within the range of 250–350 °C, and the corresponding N_2_ selectivity was ~81% [12].

In our previous work, a Pt-V tandem catalyst was synthesized by directly mixing and grinding a certain mass of the VW/TiO_2_ catalyst with a small quantity of the Pt/Al_2_O_3_ catalyst [9]. Results of the experiments indicated that NH_3_-SCO and NH_3_-SCR reactions over the mixed catalysts displayed a synergistic effect, enabling them to exhibit excellent low-temperature redox performance. In situ DRIFTS results showed that NH_3_ conversion reactions on the surface of mixed catalysts followed both the i-SCR mechanism and hydrazine mechanism. Moreover, authors synthesized a series of Pt-modified V/TiO_2_ catalysts (0.04 wt.% Pt and 0.5 wt.% V) by using the impregnation–deposition method [22]. The effect of the V and Pt deposition sequence on NH_3_-SCO performance was explored. The results displayed that V_0.5_/Pt_0.04_/TiO_2_, Pt_0.04_/V_0.5_/TiO_2_, and Pt_0.04_V_0.5_/TiO_2_ catalysts all possessed outstanding NH_3_ conversion performance. They could achieve complete NH_3_ conversion at around 250 °C. But the V_0.5_/Pt_0.04_/TiO_2_ catalyst exhibited much better N_2_ selectivity than the other two catalysts. However, the mechanism by which the V_0.5_/Pt_0.04_/TiO_2_ tandem catalyst achieves high N_2_ selectivity remains unclear. According to the i-SCR mechanism, N_2_ selectivity is mainly determined by the V component. Thus, an exploration of the modulating effect of the V loading amount on NH_3_-SCO performance may be a promising approach to revealing the mechanism of the high N_2_ selectivity of the V_0.5_/Pt/TiO_2_ tandem catalyst. But similar studies have not been reported yet.

Herein, a series of V*_x_*/Pt/TiO_2_ tandem catalysts were prepared via a two-step impregnation–deposition process. The influence of the V loading amount on the NH_3_-SCO performance of the V*_x_*/Pt/TiO_2_ catalysts was systematically investigated. The results showed that, compared with other V*_x_*/Pt/TiO_2_ catalysts, the V_0.5_/Pt/TiO_2_ catalyst exhibited outstanding comprehensive SCO performance. It could completely remove 3000 ppm of NH_3_ within the range of 250–450 °C and corresponding N_2_ selectivity was higher than 90%. Compared to previously reported NH_3_-SCO catalysts (Table S1), the V_0.5_/Pt/TiO_2_ catalyst also possessed a remarkable performance. The operating temperature window of the V_0.5_/Pt/TiO_2_ catalyst was well-matched with the range of exhaust temperatures in NH_3_-fuel engines (280–450 °C). In order to explore the mechanism underlying the excellent N_2_ selectivity of the V_0.5_/Pt/TiO_2_ catalyst, some characterization tests including N_2_ adsorption–desorption, X-ray Diffraction (XRD), X-ray Photoelectron Spectroscopy (XPS), a High-Resolution Transmission Electron Microscope (HRTEM), Energy Dispersive X-Ray Spectroscopy (EDX), O_2_ Temperature Programmed Desorption (O_2_-TPD), H_2_ Temperature-Programmed Reduction (H_2_-TPR), and NH_3_ Temperature-Programmed Desorption (NH_3_-TPD), and in situ Diffuse Reflection Infrared Fourier Transform Spectroscopy (in-situ DRIFTS) were carried out. The structure–efficiency relation and surface reaction pathways were discussed in depth based on characterization results. It is well known that for NH_3_-SCO catalysts, simultaneously achieving high NH_3_ conversion efficiency and N_2_ selectivity still remains a great challenge. This work is novel in uncovering the significant regulatory effect of V loading on N_2_ selectivity of V*_x_*/Pt/TiO_2_ catalysts, elucidating the relevant regulatory mechanism, and revealing the mechanism by which the V_0.5_/Pt/TiO_2_ catalyst achieves a high NH_3_ conversion rate and N_2_ selectivity simultaneously.

## 2. Experimental Section

### 2.1. Catalyst Preparation

Based on our previous research [22], the V*_x_*/Pt/TiO_2_ catalysts with 0.04 wt.% Pt and *x* wt.% V were prepared using a two-step impregnation–deposition method. Different mass fractions of V (*x* = 0.1, 0.3, 0.5, 0.7, 0.9 wt.%) were incorporated via impregnation in the second step. The detailed preparation processes of V*_x_*/Pt/TiO_2_ catalysts were given in the Supplementary Materials. The Pt/TiO_2_ (0.04 wt.% Pt) and V/TiO_2_ (0.5 wt.% V) catalysts were synthesized by the impregnation method for better benchmarking. The specific synthesis procedures can be found in the Supplementary Materials.

### 2.2. Catalyst Characterization

Various techniques including XRD, N_2_ adsorption–desorption, XPS, HRTEM, EDX, NH_3_-TPD, O_2_-TPD, and H_2_-TPR were employed to characterize the prepared catalysts so as to reveal the physical and chemical properties. Additionally, in situ DRIFTS was used to investigate key intermediates in NH_3_-SCO reactions. Detailed information on catalyst characterization can be found in the Supplementary Materials.

### 2.3. Catalytic Performance Test

The NH_3_-SCO performance of the V*_x_*/Pt/TiO_2_ catalysts was tested using a fixed-bed reactor (Ming Yang Company, Nanjing, China). A catalyst dosage of 0.2 mL was used in each test. Prior to testing, a N_2_ stream was used to purge the catalyst at 150 °C for 1 h, removing any residual contaminants. The catalyst was then exposed to a reactant gas flow of 200 mL/min, consisting of 3000 ppm NH_3_, 5 vol.% O_2_, and N_2_ as the balance gas. Then, reaction temperature was gradually increased from 200 to 450 **°**C at a rate of 2 **°**C/min. During this process, the concentrations of NH_3_, NO, NO_2_, and N_2_O were monitored by using a FT-IR spectrometer (IGS, ThermoFisher Scientific, Waltham, MA, USA). Based on Equations (4) and (5), the NH_3_ conversion efficiency and N_2_ selectivity could be calculated:

(4) $${\mathrm{NH}}_{3}\;\mathrm{conversion}=\frac{{\mathrm{NH}}_{3,\mathrm{in}}-{\mathrm{NH}}_{3,\mathrm{out}}}{{\mathrm{NH}}_{3,\mathrm{in}}}\times 100\%$$

(5) $$N_{2}\;\mathrm{selectivity}=\frac{{\mathrm{NH}}_{3,\mathrm{in}}-{\mathrm{NH}}_{3,\mathrm{out}}-{\mathrm{NO}}_{\mathrm{out}}-{\mathrm{NO}}_{2,\mathrm{out}}-{2\times N}_{2}O_{\mathrm{out}}}{{\mathrm{NH}}_{3,\mathrm{in}}-{\mathrm{NH}}_{3,\mathrm{out}}}\times 100\%$$

## 3. Results

### 3.1. NH_3_-SCO Performance

**Figure 1:**
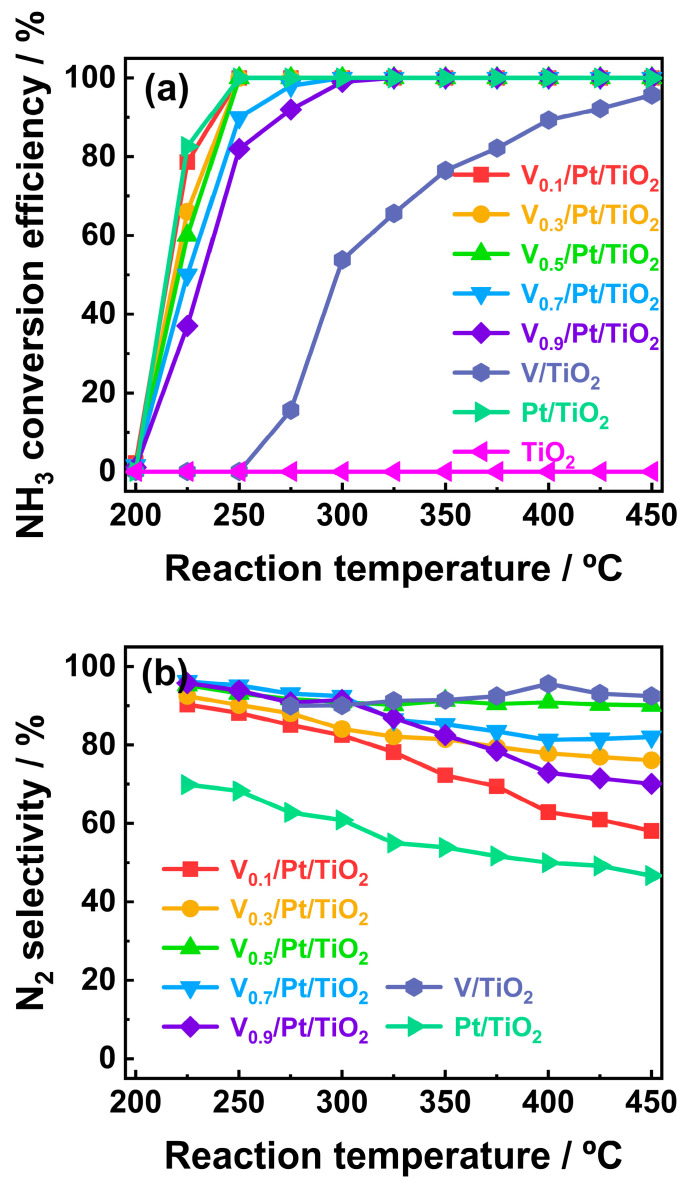
(**a**) NH_3_ conversion efficiencies and (**b**) N_2_ selectivities of prepared catalysts. **Reaction conditions:** [NH_3_]_in_ = 3000 ppm, [O_2_]_in_ = 5 vol%, Flowrate = 200 mL/min, N_2_ balance gas.

The NH_3_ conversion efficiencies of V*_x_*/Pt/TiO_2_ catalysts are illustrated in Figure 1a. It can be seen that with the reaction temperature increased from 200 to 250 °C, the NH_3_ conversion efficiencies of the V*_x_*/Pt/TiO_2_ catalysts increased significantly. When the reaction temperature was 250 °C, the NH_3_ conversion efficiencies of the V_0.1_/Pt/TiO_2_, V_0.3_/Pt/TiO_2_, and V_0.3_/Pt/TiO_2_ catalysts were 100%, while the NH_3_ conversion efficiencies of the V_0.7_/Pt/TiO_2_ and V_0.9_/Pt/TiO_2_ catalysts were 89.2% and 83.5%, respectively. When the reaction temperature was further increased from 250 to 300 °C, the NH_3_ conversion efficiencies of the V_0.7_/Pt/TiO_2_ and V_0.9_/Pt/TiO_2_ catalysts reached 100%. The above results indicate that, compared to V_0.7_/Pt/TiO_2_ and V_0.9_/Pt/TiO_2_ catalysts, the V_0.1_/Pt/TiO_2_, V_0.3_/Pt/TiO_2_, and V_0.5_/Pt/TiO_2_ catalysts exhibit better NH_3_ catalytic conversion performance. 

The N_2_ selectivity of the V*_x_*/Pt/TiO_2_ catalysts within the temperature range of 200–450 °C is shown in Figure 1b. It can be observed that when the loading amount of V was increased from 0.1 wt.% to 0.5 wt.%, the N_2_ selectivities of the V*_x_*/Pt/TiO_2_ catalysts were distinctly enhanced. It is noteworthy that, compared with other V*_x_*/Pt/TiO_2_ catalysts, the V_0.5_/Pt/TiO_2_ catalyst exhibited outstanding N_2_ selectivity. The N_2_ selectivity of the V_0.5_/Pt/TiO_2_ catalyst exceeded 90% across the entire temperature range. The above results imply that a moderate increase in the loading amount of V might be beneficial for enhancing NH_3_-SCR reactions over V*_x_*/Pt/TiO_2_ catalysts, thereby promoting the generation of N_2_ via Equations (3), (6) and (7)

(6) $$2NH_{3}+\mathrm{NO}+{\mathrm{NO}}_{2}\to {2N}_{2}+{3H}_{2}O$$

(7) $$N_{2}O+2NH_{3}+O_{2}\to {2N}_{2}+{3H}_{2}O$$

When the V loading amount was further increased from 0.5 wt.% to 0.9 wt.%, the N_2_ selectivity of the V*_x_*/Pt/TiO_2_ catalysts gradually decreased. According to previous studies [22,23,24], this might be related to the excessive catalytic oxidation of NH_3_ by the V species.

The NH_3_-SCO performance of Pt/TiO_2_ (0.04 wt.% Pt) and V/TiO_2_ (0.5 wt.% V) catalysts and the bare TiO_2_ support were evaluated for benchmarking. The results are shown in Figure 1. It could be seen that the Pt/TiO_2_ catalyst exhibited excellent NH_3_ conversion performance. It could achieve complete conversion of 3000 ppm NH_3_ within the temperature range of 250–450 °C. Its NH_3_ conversion performance was close to that of the V_0.1_/Pt/TiO_2_, V_0.3_/Pt/TiO_2_, and V_0.5_/Pt/TiO_2_ catalysts, and was significantly better than that of the V_0.7_/Pt/TiO_2_ and V_0.9_/Pt/TiO_2_ catalysts. But the N_2_ selectivity of the Pt/TiO_2_ catalyst within the above temperature range was lower than 70%, which was significantly lower than that of the V*_x_*/Pt/TiO_2_ catalysts. Compared with the V*_x_*/Pt/TiO_2_ and Pt/TiO_2_ catalysts, the NH_3_ conversion performance of the V/TiO_2_ catalyst was relatively poor. It only shows the obvious NH_3_ conversion performance when the reaction temperature was higher than 250 °C. However, the V/TiO_2_ catalyst exhibited excellent N_2_ selectivity, which exceeded 90% within the temperature range of 275–450 °C. The bare TiO_2_ support did not show the catalytic activity within the whole temperature range.

**Figure 2:**
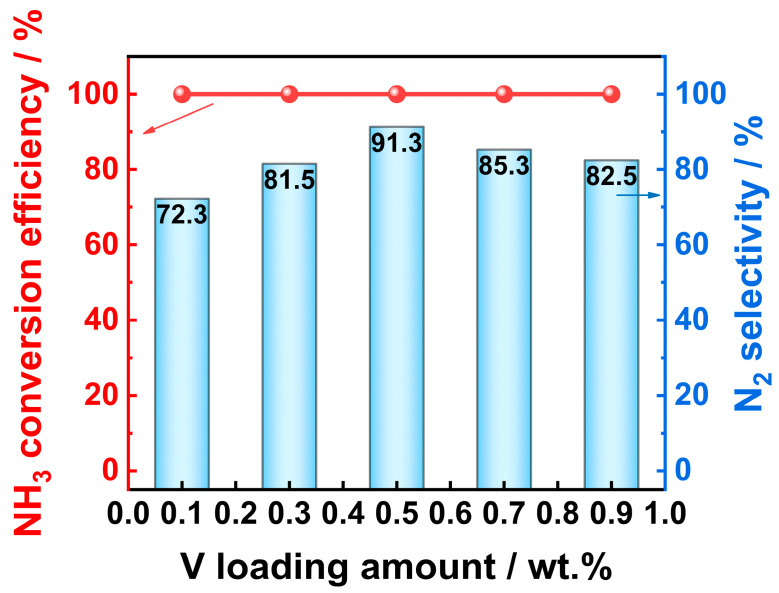
Change in NH_3_-SCO performance of V*_x_*/Pt/TiO_2_ catalysts with V loading amount. **Reaction conditions:** [NH_3_]_in_ = 3000 ppm, [O_2_]_in_ = 5 vol%, Flowrate = 200 mL/min, N_2_ balance gas.

In order to more intuitively display the influence of the V loading amount on the performance of the V*_x_*/Pt/TiO_2_ catalyst, a summary schematic comparing the catalyst performance versus the V loading amount was drawn, which is shown in Figure 2. The reaction temperature corresponding to the schematic is 350 °C. As shown in Figure 2, with the V loading amount increased from 0.1 wt.% to 0.5 wt.%, the N_2_ selectivity of the V*_x_*/Pt/TiO_2_ catalysts significantly improved from 72.3% to 91.3%. When the V loading amount further increased from 0.5 wt.% to 0.9 wt.%, the N_2_ selectivity of the V*_x_*/Pt/TiO_2_ catalyst gradually decreased from 91.3% to 82.5%. During the process of increasing the V loading amount from 0.1 wt.% to 0.9 wt.%, the NH_3_ conversion efficiency of the V*_x_*/Pt/TiO_2_ catalysts always remains at 100%. The above results indicated that, in this work, the V_0.5_/Pt/TiO_2_ catalyst with a V loading amount of 0.5 wt.% exhibited the best comprehensive SCO performance.

The NH_3_-SCO experimental results demonstrated that, compared with other V*_x_*/Pt/TiO_2_ catalysts, the V_0.5_/Pt/TiO_2_ catalyst exhibited excellent N_2_ selectivity. In comparison with the NH_3_-SCO catalysts reported previously (Table S1), the performance of the V_0.5_/Pt/TiO_2_ catalyst was also quite outstanding. Therefore, the V_0.5_/Pt/TiO_2_ catalyst was selected for further study.

In order to evaluate the repeatability of the catalyst synthesis, three batches of the V_0.5_/Pt/TiO_2_ catalyst were, respectively, synthesized using the same process, and performance tests were carried out. The results are shown in Figure S1. The data of Test 1 in Figure S1 are the performance test data of the V_0.5_/Pt/TiO_2_ catalyst mentioned in the manuscript. The data of Test 2 and Test 3 are the performance test data of the other two batches of the V_0.5_/Pt/TiO_2_ catalyst synthesized under the same conditions. It can be seen from Figure S1 that the NH_3_ conversion performances of the three batches of the V_0.5_/Pt/TiO_2_ catalyst in the temperature range of 200–450 °C are quite similar, and so is the N_2_ selectivity. This indicated that the catalyst synthesis process has high reproducibility.

**Figure 3:**
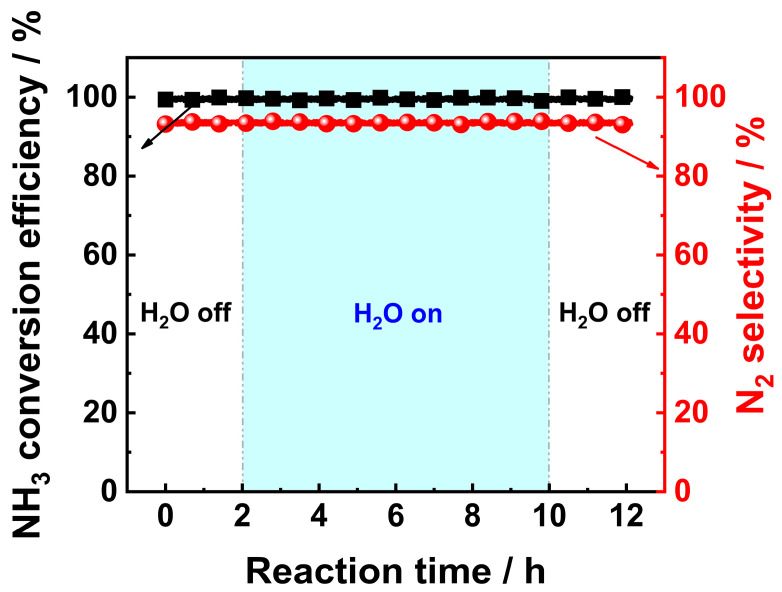
H_2_O resistance test results of V_0.5_/Pt/TiO_2_ catalyst. **Reaction conditions:** [NH_3_]_in_ = 3000 ppm, [O_2_]_in_ = 5 vol%, [H_2_O]_in_ = 10 vol.%, Flowrate = 200 mL/min, N_2_ balance gas.

The common causes of catalyst aging or deactivation after long-term use include sintering of active components, changes in structure of active components, collapse of pore structure, and catalyst poisoning caused by external impurities [24,25,26]. The humid conditions may promote aging or deactivation of catalysts. This is because under certain conditions, H_2_O molecules may react with active components, thus resulting in a reduction in catalytic activity or even deactivation [25,27]. H_2_O molecules may also react with supports of the catalyst, resulting in collapse of the pore structure, thus leading to a decline in catalytic activity [28]. Since the combustion of NH_3_ in NH_3_-fuel engines would produce H_2_O vapor, it is advisable to conduct a preliminary test to obtain a brief understanding of the H_2_O resistance of the V_0.5_/Pt/TiO_2_ catalyst. The H_2_O resistance test was carried out under the following conditions: the concentration of the H_2_O vapor was 10 vol.%, the reaction temperature was set at 300 °C, and the total experimental duration was 8 h. The results are shown in Figure 3. It can be observed that the introduction of water vapor has no significant impact on the NH_3_ conversion rate and N_2_ selectivity of the V_0.5_/Pt/TiO_2_ catalyst, indicating that the catalyst possessed good water resistance.

For the purpose of investigating the mechanism for outstanding N_2_ selectivity of the V_0.5_/Pt/TiO_2_ catalyst, some characterization techniques have been employed to characterize physicochemical properties of V*_x_*/Pt/TiO_2_ catalysts. The results of the catalyst characterization tests will be presented below, and they will be discussed in detail.

### 3.2. N_2_ Adsorption and Desorption

**Figure 4:**
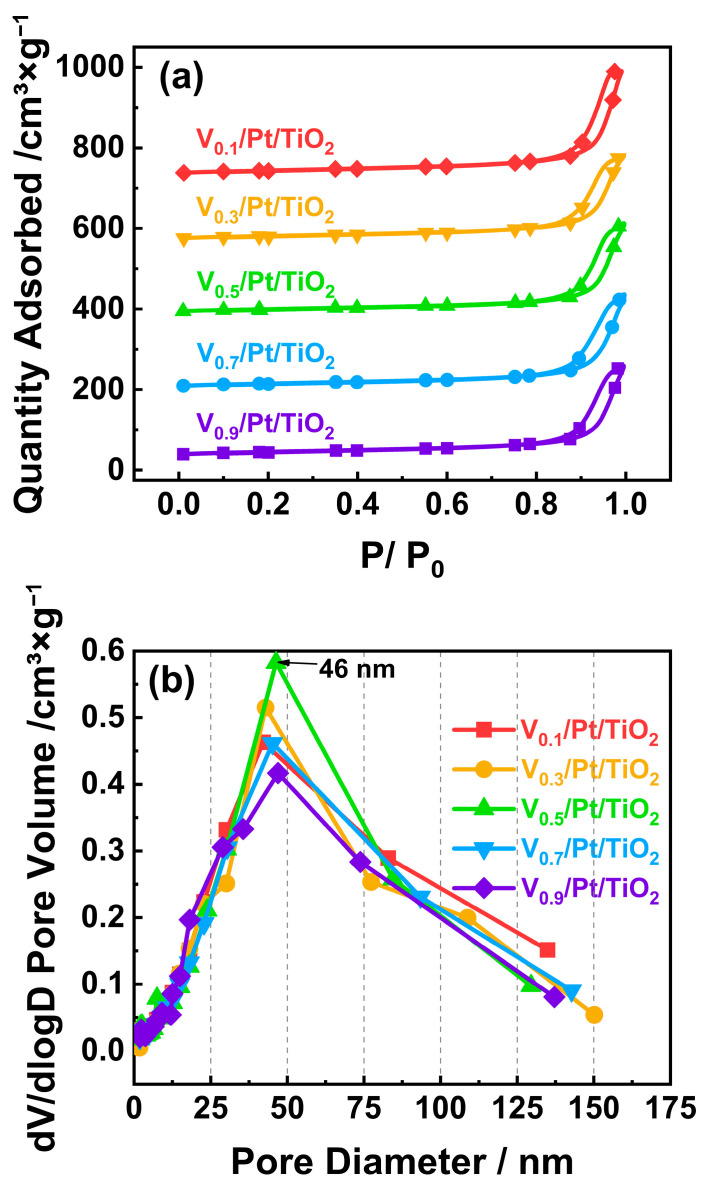
(**a**) N_2_ adsorption–desorption isotherms and (**b**) pore size distribution curves of V*_x_*/Pt/TiO_2_ catalysts.

The specific surface area of a catalyst is usually closely related to the adsorption of reactants and the dispersion of active species on the catalyst surface, thereby having a significant impact on the performance of the catalyst [20,21]. Generally, a high specific surface area of the catalyst is conducive to adsorbing more NH_3_ and O_2_, which is beneficial for promoting NH_3_-SCO performance [22,23]. A high specific surface area of the catalyst is also conducive to active species being more evenly dispersed on the catalyst surface, thereby enriching active sites and improving the NH_3_-SCO performance [24]. To characterize the surface physical properties of the V*_x_*/Pt/TiO_2_ catalysts, N_2_ adsorption and desorption experiments were performed, and the results are presented in Figure 4. Figure 4a shows the N_2_ adsorption–desorption isotherms of the V*_x_*/Pt/TiO_2_ catalysts. According to previous studies, the isotherms could be categorized as type IV(a) with H3 hysteresis loops, which implied the existence of a large number of mesopores on the surface of the tested catalysts [22,23,24]. It can be seen from Figure 4b that the surface pore sizes of the V*_x_*/Pt/TiO_2_ catalysts were predominantly around 46 nm, which is in agreement with the results shown in Figure 4a. It can also be observed from Figure 4b that, compared with other V*_x_*/Pt/TiO_2_ catalysts, the V_0.5_/Pt/TiO_2_ catalyst possessed more abundant 45 nm pores on the surface. As shown in Table S2, the specific surface area of the V_0.5_/Pt/TiO_2_ catalyst was higher than those of other V*_x_*/Pt/TiO_2_ catalysts. But there was little difference in surface pore volume and pore diameter between the V_0.5_/Pt/TiO_2_ catalyst and each of the other V*_x_*/Pt/TiO_2_ catalysts. The results shown in Figure 4b and Table S2 indicate that 0.5 wt.% of the V loading amount might be conducive to the formation of mesopores on the surface of the V_0.5_/Pt/TiO_2_ catalyst, thus resulting in a relatively higher specific surface area of the V_0.5_/Pt/TiO_2_ catalyst. Generally, a higher specific surface area is conducive to the dispersion of active metal components and the adsorption of reactants on the catalyst surface, thus improving the catalytic activity [29]. Thus, the relatively larger specific surface area of the V_0.5_/Pt/TiO_2_ catalyst contributed to its excellent NH_3_-SCO performance.

### 3.3. XRD

**Figure 5:**
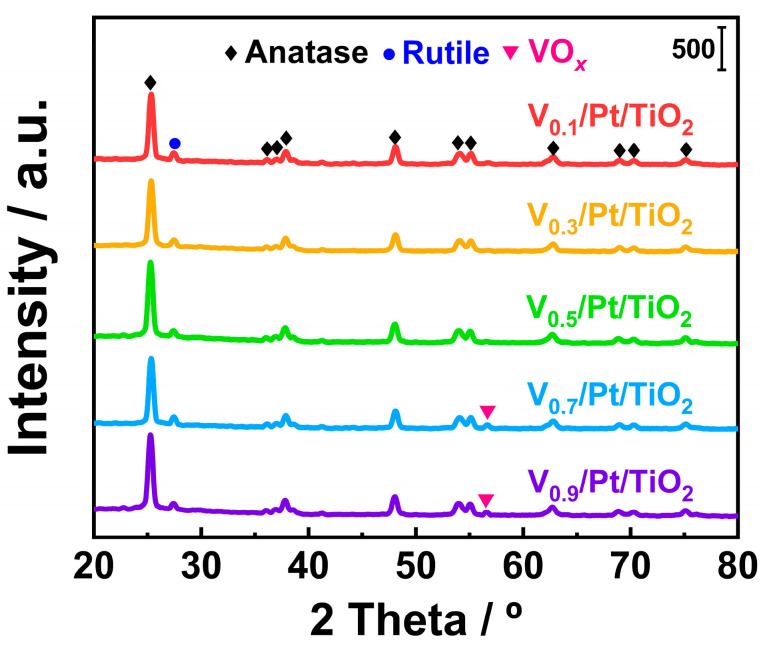
XRD spectra of V*_x_*/Pt/TiO_2_ catalysts.

The crystal structures of active species and support are closely related to the performance of the catalyst. The XRD technique is considered a common technique for obtaining the above information. XRD tests were conducted on the V*_x_*/Pt/TiO_2_ catalyst, and the results are presented in Figure 5. Several obvious diffraction peaks can be observed in the XRD spectra of the V*_x_*/Pt/TiO_2_ catalysts. Diffraction peaks centered at 25.0°, 36.6°, 38.0°, 38.8°, 48.1°, 54.3°, 55.2°, 62.8°, 68.9°, 70.6°, and 75.3° could be attributed to anatase TiO_2_ phases (ICDD #21-1276) [9,22,23]. The diffraction peak centered at 27.5° could be attributed to rutile TiO_2_ phases (ICDD #21-1272) [24]. It can be seen from Figure 5 that there were no peaks attributed to the VO*_x_* phase in the XRD spectra of the V_0.1_/Pt/TiO_2_, V_0.3_/Pt/TiO_2_, and V_0.5_/Pt/TiO_2_ catalysts. This suggested that the VO*_x_* species over the above three catalysts were in a highly dispersed state or an amorphous state. As shown in Figure 5, a small diffraction peak (56.8°) emerged in each of the XRD spectra of the V_0.7_/Pt/TiO_2_ and V_0.9_/Pt/TiO_2_ catalysts. These peaks were ascribed to the VO*_x_* species (ICDD #19-1401), indicating that there were tiny VO*_x_* crystals over the V_0.7_/Pt/TiO_2_ and V_0.9_/Pt/TiO_2_ catalysts [22,23,24]. The above results show that when the V loading amounts were 0.1 wt.%, 0.3 wt.%, and 0.5 wt.%, the VO*_x_* species could be well dispersed on the catalyst surface. When the V loading amounts were 0.7 and 0.9 wt.%, the VO*_x_* species on the surface of the catalyst agglomerated slightly. It can be seen from Figure 5 that no diffraction peaks that could be attributed to the Pt species appeared in the XRD spectra of the V*_x_*/Pt/TiO_2_ catalysts. This phenomenon could be primarily ascribed to the extremely low Pt contents in the V*_x_*/Pt/TiO_2_ catalysts.

### 3.4. XPS

**Figure 6:**
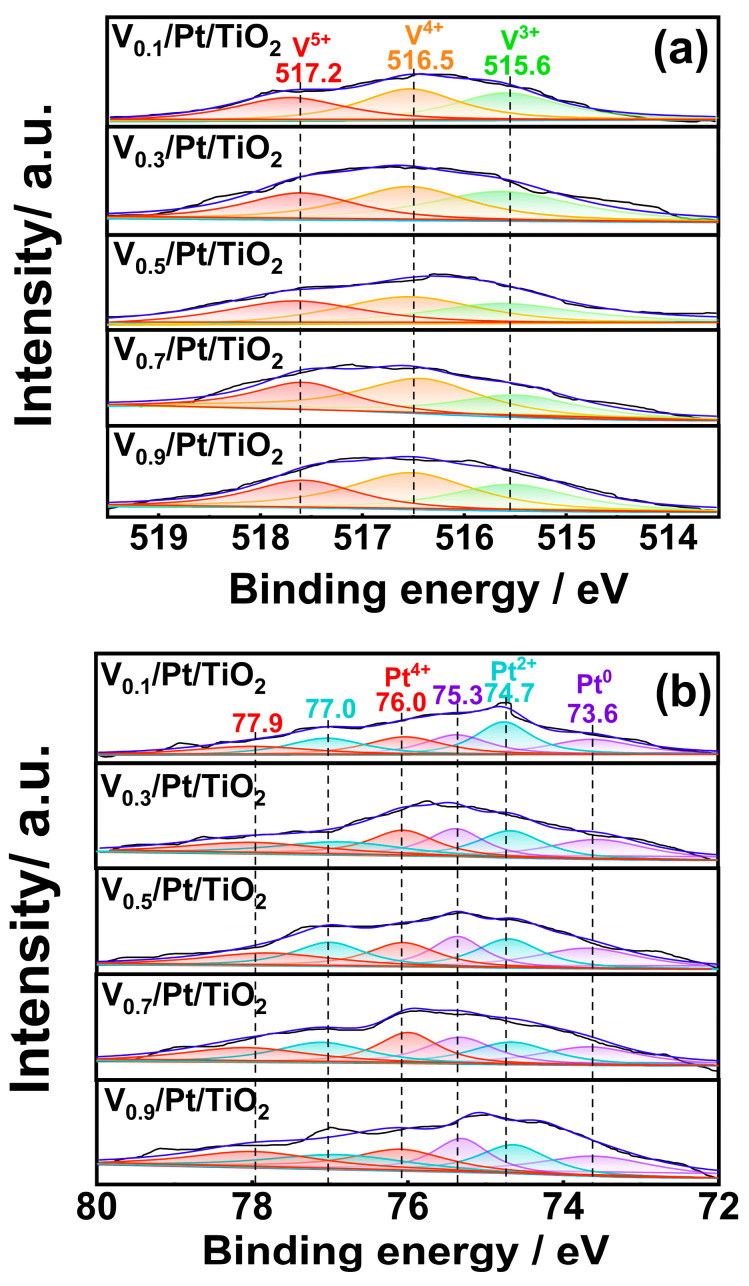

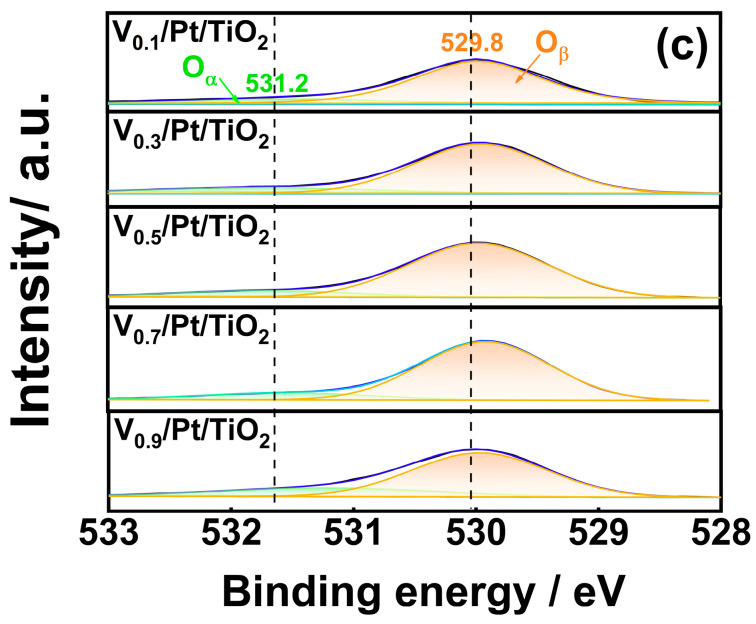
(**a**) V 2p, (**b**) Pt 4f, (**c**) O 1s XPS spectra of V*_x_*/Pt/TiO_2_ catalysts.

The NH_3_-SCO performance of the catalyst is greatly influenced by the valence states and relative contents of the elements on the surface. XPS experiments were performed to analyze the valence states and relative contents of the main elements over the V*_x_*/Pt/TiO_2_ catalysts. The test results are presented in Figure 6 and Table S3. As shown in Figure 6a, the V 2p spectrum of each V*_x_*/Pt/TiO_2_ catalyst could be split into three sub-peaks. Sub-peaks centered at 515.6, 516.5, and 517.2 eV were attributable to the V^3+^, V^4+^, and V^5+^ species, respectively [22,23,24]. As shown in Table S3, with the V loading amount increasing from 0.1 wt.% to 0.5 wt.%, the V^5+^/(V^3+^+V^4+^+V^5+^) ratios of the V*_x_*/Pt/TiO_2_ catalysts increased from 28.20% to 32.65%. When the V loading amount was further increased from 0.5 wt.% to 0.9 wt.%, the V^5+^/(V^3+^+V^4+^+V^5+^) ratios of the V*_x_*/Pt/TiO_2_ catalysts gradually decreased from 32.65% to 25.22%. It has been reported that the V^5+^ species possesses good NH_3_-SCR catalytic activity [24]. Thus, the relatively high V^5+^/(V^3+^+V^4+^+V^5+^) ratio of the V_0.5_/Pt/TiO_2_ catalyst was conducive to promoting the generation of N_2_, enabling the V_0.5_/Pt/TiO_2_ catalyst to exhibit excellent N_2_ selectivity.

The Pt 4f spectra of the V*_x_*/Pt/TiO_2_ catalyst are presented in Figure 6b. It can be seen that each of spectra could be resolved into four sub-peaks. As marked in Figure 6b, sub-peaks in the range of 73.6 to 77.9 eV could be ascribed to Pt^0^, Pt²⁺, and Pt⁴⁺ species [27,28]. As shown in Table S3, when the V loading amount was increased from 0.1 wt.% to 0.5 wt.%, the Pt^0^/(Pt^0^+Pt^2+^+Pt^4+^) ratio of the V*_x_*/Pt/TiO_2_ catalyst increased from 35.56% to 37.66%. When the V loading amount was further increased from 0.5 wt.% to 0.9 wt.%, the Pt^0^/(Pt^0^+Pt^2+^+Pt^4+^) ratio of the V*_x_*/Pt/TiO_2_ catalyst decreased from 37.66% to 35.23%. This suggested that adopting a V loading amount of 0.5 wt.% was conducive to the generation of the Pt^0^ species. Compared to the Pt^2+^ and Pt^4+^ species, the Pt^0^ species possess much higher catalytic oxidation activity, which is mainly attributed to its high activity in O_2_ dissociation [22,23]. Therefore, the existence of a relatively larger amount of Pt^0^ species was beneficial for the V_0.5_/Pt/TiO_2_ catalyst to oxidize more NH_3_ into NO*_x_*, providing more reactants for the NH_3_-SCR reaction, thus promoting the generation of N_2_.

Figure 6c displays the O 1s spectra of the V*_x_*/Pt/TiO_2_ catalysts. It can be seen that the spectrum of each V*_x_*/Pt/TiO_2_ catalyst could be resolved into two sub-peaks. Sub-peaks centered at 531.2 eV and 529.8 eV could be ascribed to chemisorbed oxygen species (O_α_) and lattice oxygen species (O_β_), respectively. Table S3 shows that the O_α_/(O_α_+O_β_) ratio of the V_0.5_/Pt/TiO_2_ catalyst is 15.23%, which is higher than those of other V*_x_*/Pt/TiO_2_ catalysts. Compared with O_β_ species, the O_α_ species possess higher activity in the NH_3_ oxidation reaction [29,30,31,32]. Thus, a relatively larger amount of O_α_ species was conducive to oxidation of more NH_3_ into NO*_x_* and N_2_. Subsequently, NO*_x_* could be converted into N_2_ through NH_3_-SCR reactions, enabling the V_0.5_/Pt/TiO_2_ catalyst to exhibit a good N_2_ selectivity.

### 3.5. O_2_-TPD

**Figure 7:**
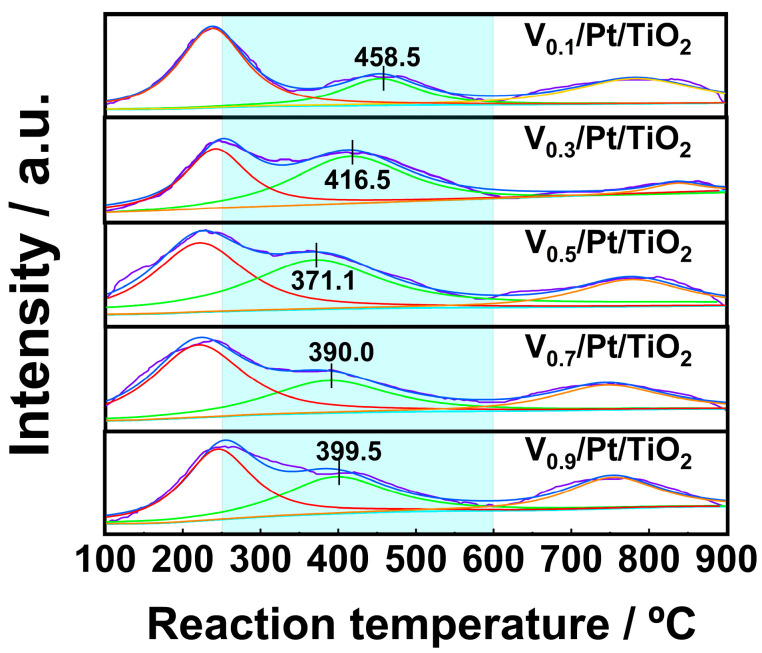
O_2_-TPD profiles of V*_x_*/Pt/TiO_2_ catalysts.

The surface oxygen species can participate in NH_3_ catalytic oxidation reactions, thereby influencing the SCO performance of the catalysts. For the purpose of characterizing the active oxygen species over the V*_x_*/Pt/TiO_2_ catalysts, O_2_-TPD experiments were performed, and the results are shown in Figure 7. Previous studies pointed out that O_2_ desorption below 250 °C mainly resulted from the desorption of physically adsorbed oxygen [9,22,23]. O_2_ desorption between 250 °C and 600 °C was mainly caused by the desorption of chemically adsorbed oxygen from oxygen vacancies. Desorption of O_2_ between 600 °C and 900 °C was mainly related to the desorption of surface lattice oxygen.

It can be seen from Figure 7 that for the O_2_-TPD profile of each V*_x_*/Pt/TiO_2_ catalyst, there is a single peak within the range of 100–250 °C, which implies that a great amount of physically adsorbed oxygen desorbed from the surface of the V*_x_*/Pt/TiO_2_ catalyst in this temperature range. As shown in Figure 7, each of the O_2_-TPD profiles of the V*_x_*/Pt/TiO_2_ catalysts had one peak in the range of 250 to 600 °C. The peak of the V_0.5_/Pt/TiO_2_ catalyst within the above temperature range was centered at 371.1 °C, which was significantly lower than the peak temperatures of the other V*_x_*/Pt/TiO_2_ catalysts. This indicates that, compared with other V*_x_*/Pt/TiO_2_ catalysts, the oxygen vacancies on the surface of the V_0.5_/Pt/TiO_2_ catalyst exhibit higher activity within the range of 250 to 600 °C [33]. This was beneficial for the V_0.5_/Pt/TiO_2_ catalyst to adsorb and activate O_2_, enabling it to exhibit excellent NH_3_-SCO performance.

### 3.6. Redox Performance

**Figure 8:**
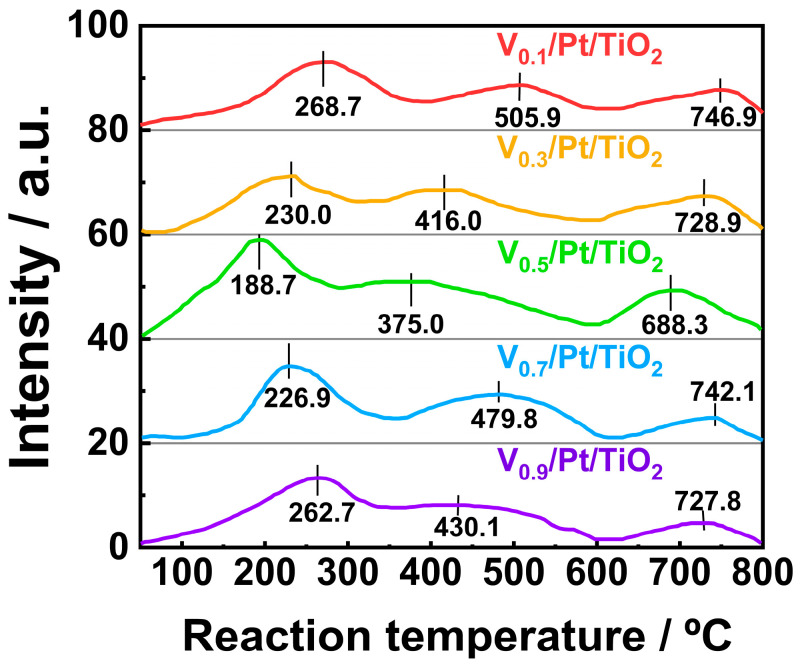
H_2_-TPR profiles of V*_x_*/Pt/TiO_2_ catalysts.

The NH_3_-SCO performance of the catalysts is strongly associated with their redox performance. In order to investigate the redox performance of the V*_x_*/Pt/TiO_2_ catalysts, H_2_-TPR tests were carried out over a temperature range of 100 to 800 °C, and the results are shown in Figure 8. The H_2_-TPR profiles of each V*_x_*/Pt/TiO_2_ catalyst contained three reduction peaks. The reduction peaks at 268.7, 230.0, 188.7, 226.9, and 262.7 °C were attributed to the reduction processes of the Pt^2+^ and V^5+^ species. During these processes, Pt^2+^ was reduced to Pt^0^ and V^5+^ was reduced to V^4+^ [31,32,33,34]. The reduction processes of the V^4+^ species to the V^3+^ species and subsequent reduction in the V^3+^ species accounted for reduction peaks at 505.9, 416.0, 375.0, 479.8, and 430.1 °C [31,32,33,34,35]. As TiO_2_ was difficult to react with H_2_ under 600 °C, reduction peaks at 746.9, 728.9, 688.3, 742.1, and 727.8 °C were attributed to the reduction in the TiO_2_ species [34].

It is worth noting that the temperatures corresponding to three reduction peaks in the H_2_-TPR profile of the V_0.5_/Pt/TiO_2_ catalyst were lower than those of the other V*_x_*/Pt/TiO_2_ catalysts. This might be ascribed to the strong interaction between the active components and TiO_2_ support [36]. The above results indicate that throughout the entire temperature, the V_0.5_/Pt/TiO_2_ catalyst exhibited better redox performance compared to other V*_x_*/Pt/TiO_2_ catalysts. In addition, as shown in Table S4, the H_2_ consumption value (0.75 mmol/g) of the V_0.5_/Pt/TiO_2_ catalyst was higher than those of other V*_x_*/Pt/TiO_2_ catalysts. This indicated that compared with other V*_x_*/Pt/TiO_2_ catalysts, there were more abundant reducible species on the surface of the V_0.5_/Pt/TiO_2_ catalyst. Generally, outstanding redox performance and abundant reducible species are beneficial for the catalyst to exhibit excellent activity in the NH_3_-SCR reaction. Thus, the V_0.5_/Pt/TiO_2_ catalyst could achieve higher N_2_ selectivity via the i-SCR mechanism.

### 3.7. NH_3_-TPD

**Figure 9:**
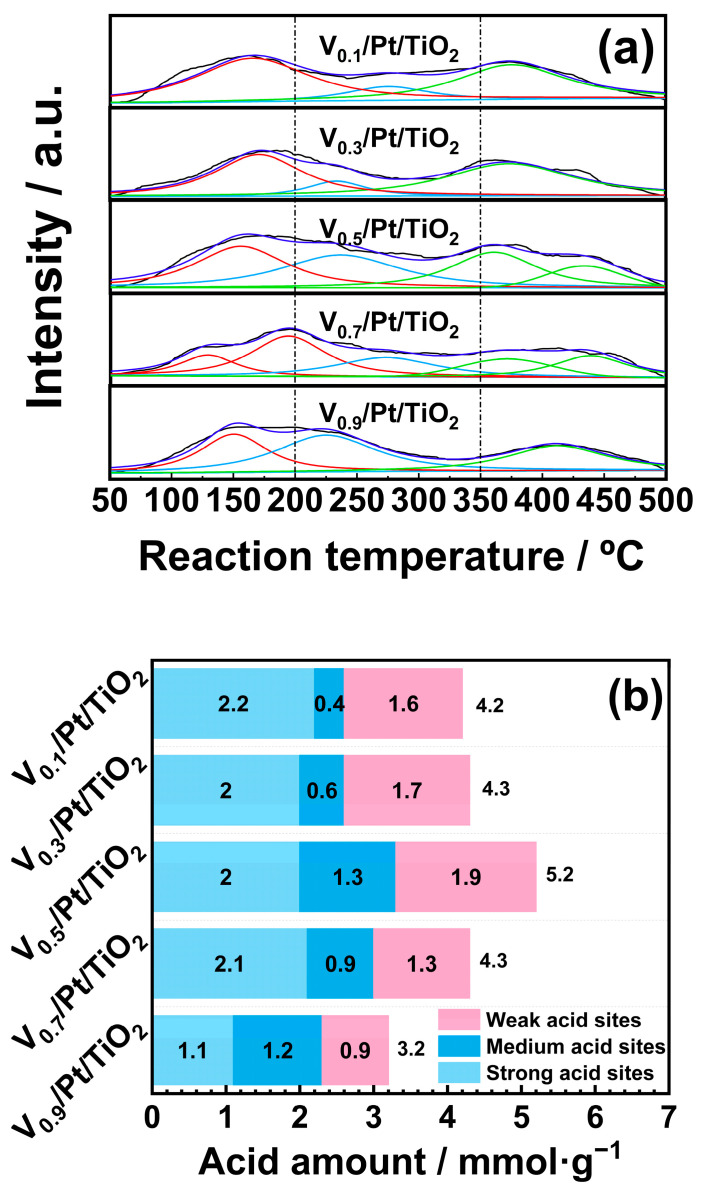
(**a**) NH_3_-TPD profiles and (**b**) acid sites quantities of V*_x_*/Pt/TiO_2_ catalysts.

Surface acidity played a pivotal role in facilitating adsorption and activation of NH_3_ on the catalyst surface, which was critical for the NH_3_-SCO reaction. The surface acidity of the V*_x_*/Pt/TiO_2_ catalysts was tested through NH_3_-TPD experiments, and the results are displayed in Figure 9. Figure 9a displays the NH_3_-TPD profiles of the V*_x_*/Pt/TiO_2_ catalysts. Based on previous studies, the NH_3_-TPD profiles in the temperature range of 50 to 200 °C were ascribed to NH_3_ desorption from weak acid sites. NH_3_-TPD profiles between 200 and 350 °C were attributed to the desorption of NH_3_ from medium acid sites [36,37,38]. The NH_3_-TPD profiles in the temperature range of 350 to 500 °C were ascribed to the NH_3_ desorption from strong acid sites [39,40,41]. The quantities of acid sites on the surface of V*_x_*/Pt/TiO_2_ catalysts were calculated by integrating the areas of sub-peaks in NH_3_-TPD profiles, and the results are shown in Figure 9b. It can be seen that with the V loading amount increasing from 0.1 wt.% to 0.5 wt.%, the total acid quantities of V*_x_*/Pt/TiO_2_ catalysts gradually increased from 4.2 to 5.2 mmol/g. When the V loading amount was further increased from 0.5 wt.% to 0.9 wt.%, the total acid quantities of V*_x_*/Pt/TiO_2_ catalysts decreased from 5.2 to 3.2 mmol/g. This suggested that compared with other V*_x_*/Pt/TiO_2_ catalysts, the V_0.5_/Pt/TiO_2_ catalyst had more abundant acid sites on its surface. It can be seen from Figure 9b that the quantities of the weak and medium acid sites on the surface of the V_0.5_/Pt/TiO_2_ catalyst were higher than those of other V*_x_*/Pt/TiO_2_ catalysts. This was conducive to the adsorption and activation of NH_3_ on the surface of the V_0.5_/Pt/TiO_2_ catalyst [34,35,36,37,38,39,40,41,42,43,44,45,46], enabling the V_0.5_/Pt/TiO_2_ catalyst to exhibit excellent N_2_ selectivity.

### 3.8. HRTEM and EDX

**Figure 10:**
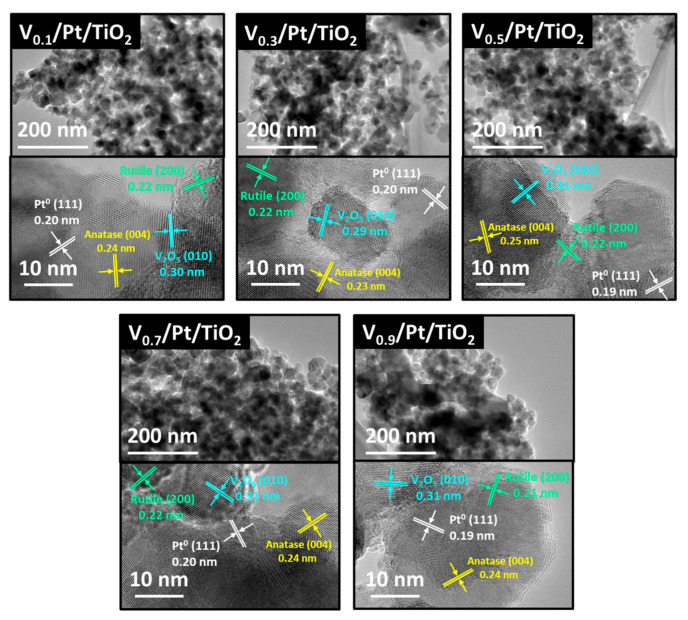
HRTEM images of V*_x_*/Pt/TiO_2_ catalysts.

**Figure 11:**
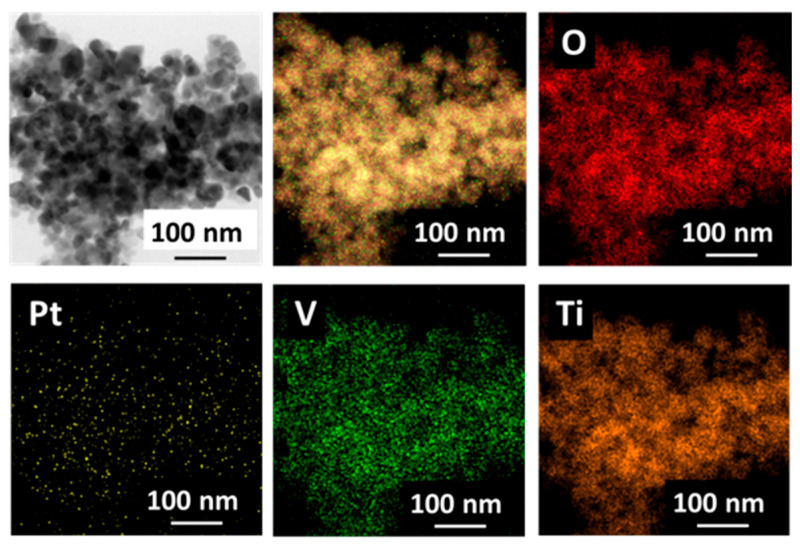
EDX mappings of V_0.5_/Pt/TiO_2_ catalyst.

HRTEM tests were conducted on V*ₓ*/Pt/TiO_2_ catalysts, and EDX tests were carried out on the V_0.5_/Pt/TiO_2_ catalyst. The results are shown in Figure 10 and Figure 11. It can be seen from Figure 10 that the V*_x_*/Pt/TiO_2_ catalysts all exhibited a granular microscopic morphology, with a particle size of approximately 20–50 nm. This was mainly because in this work, P25 TiO_2_ nanoparticles were used as the catalyst support. It could be observed that distinct lattice fringes existed in the highly magnified images. As marked in Figure 10, these fringes could be attributed to anatase TiO_2_ species (ICDD 21-1276), rutile TiO_2_ species (ICPD 21-1272), the Pt species (ICDD 43-1100), and the V_2_O_5_ species (ICDD 19-1401) [27,46]. This indicated that there were nanocrystals of the above-mentioned species on the surface of the catalysts.

The EDX test was employed to characterize the dispersion state of O, Pt, V, and Ti elements on the surface of the V_0.5_/Pt/TiO_2_ catalyst, and the results are shown in Figure 11. The results suggested that O, Pt, V, and Ti elements were uniformly distributed on the surface of the V_0.5_/Pt/TiO_2_ catalyst. Pt and V elements were in intimate contact with one another, which contributed to facilitating the interaction between the Pt and V species.

### 3.9. In Situ DRIFTS

**Figure 12:**
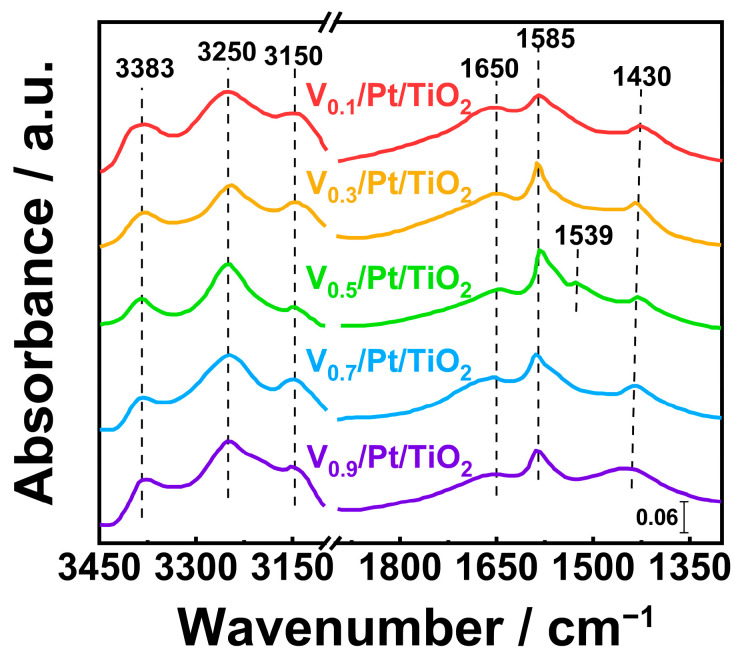
In situ DRIFT spectra of NH_3_ and O_2_ co-adsorbed over V*_x_*/Pt/TiO_2_ catalysts at 350 °C for 30 min.

The surface reaction pathways of NH_3_-SCO catalysts are closely related to their N_2_ selectivity. With the aim of exploring reaction pathways over V*_x_*/Pt/TiO_2_ catalysts, in situ DRIFTS experiments were carried out for the reaction between NH_3_ and O_2_ at 350 °C, and the results are shown in Figure 12. The results of in situ DRIFTS experiments have been discussed as follows. Detailed information about in situ DRIFTS experiments is presented in the Supplementary Materials.

As shown in Figure 12, the NH_3_ species (3383, 3150 cm*^−^*^1^), NH_3_ species coordinated at Lewis acid sites (3250 cm*^−^*^1^), NO_2_ species (1650 cm*^−^*^1^), bidentate nitrate species (1585 cm*^−^*^1^), and NH_4_^+^ species coordinated at Brønsted acid sites (1436 cm*^−^*^1^) on the surface of V*_x_*/Pt/TiO_2_ catalysts were capable of taking part in the NH_3_-SCO reaction [47,48,49,50,51,52,53]. It can be seen from Figure 12 that when both NH_3_ and O_2_ were introduced into reaction cell, a distinct signal of -NH_2_ species (1539 cm*^−^*^1^) appeared in the in situ DRIFTS spectrum of the V_0.5_/Pt/TiO_2_ catalyst [35]. But during the same reaction process, the signal of the -NH_2_ species did not appear in the in situ DRIFTS spectra of other V*_x_*/Pt/TiO_2_ catalysts. This implied that the -NH_2_ species could be involved in the NH_3_-SCO reaction over the V_0.5_/Pt/TiO_2_ catalyst. Since the -NH_2_ species was a key intermediate in the i-SCR mechanism [53,54], the appearance of the -NH_2_ species over the V_0.5_/Pt/TiO_2_ catalyst might be associated with the promotion of the i-SCR reaction. This facilitated the formation of N_2_, consequently enhancing the N_2_ selectivity.

## 4. Discussion

**Figure 13:**
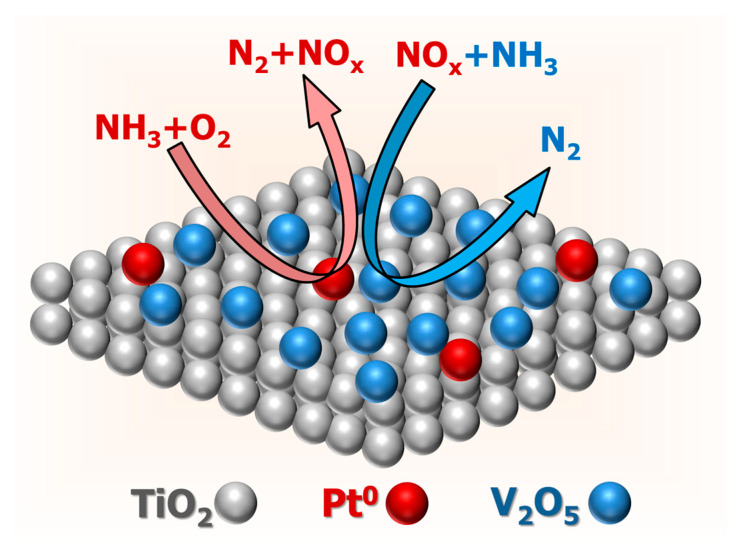
Schematic diagram of reaction pathways on the surface of V*_x_*/Pt/TiO_2_ catalysts.

HRTEM, EDX, XRD, XPS, and H_2_-TPR results indicated that there were Pt^0^ and V_2_O_5_ nanocrystals on the surface of the V_0.5_/Pt/TiO_2_ catalyst. The Pt^0^ species had a strong ability for catalytic oxidation of NH_3_. V_2_O_5_ species possessed remarkable catalytic performance for NH_3_-SCR reactions. Thus, both NH_3_-SCO and NH_3_-SCR reactions existed on the surface of the V*ₓ*/Pt/TiO_2_ catalyst, as shown in Figure 13. This enabled NH_3_ to be converted into N_2_ through the i-SCR mechanism. XPS and H_2_-TPR results implied that the valence states of the V and Pt species could be regulated by varying the V loading amount, thereby regulating the synergistic effect between NH_3_-SCO and NH_3_-SCR reactions. When the V loading amount was 0.5 wt.%, there were abundant Pt⁰ and V⁵⁺ species on the surface of the V_0.5_/Pt/TiO_2_ catalyst. This was beneficial for making the synergistic effect between NH_3_-SCO and NH_3_-SCR reactions more efficient, thus enabling the V_0_._5_/Pt/TiO_2_ catalyst to exhibit excellent N_2_ selectivity. N_2_ adsorption–desorption, NH_3_-TPD, O_2_-TPD, and in situ DRIFTS results suggested that the V_0.5_/Pt/TiO_2_ catalyst possessed a higher specific surface area, abundant surface acid sites, and oxygen vacancies, as well as better reaction pathways. This also contributed to enabling the V_0.5_/Pt/TiO_2_ catalyst to exhibit excellent N_2_ selectivity.

Due to the limitation of the article length, a systematic exploration of hydrothermal aging characteristics of the V_0.5_/Pt/TiO_2_ catalyst has not been carried out in this work. In future work, the NH_3_-SCO performance of the V_0.5_/Pt/TiO_2_ catalyst under different hydrothermal aging conditions will be systematically explored. The influence mechanism of the hydrothermal aging process on the catalytic performance will be analyzed, and corresponding strategies for improving the catalytic performance will be proposed.

## 5. Conclusions

In this work, the V*_x_*/Pt/TiO_2_ tandem catalysts were synthesized for SCO of NH_3_ by the two-step impregnation–deposition method. The synergistic effect of NH_3_-SCO and NH_3_-SCR reactions over V*_x_*/Pt/TiO_2_ tandem catalysts could be regulated by changing the V loading amount, thereby modulating the N_2_ selectivity. When the Pt loading was 0.5 wt.%, there were relatively abundant Pt^0^ species and V^5+^ species on the surface of the V_0.5_/Pt/TiO_2_ catalyst. These two species, respectively, possessed excellent NH_3_ catalytic oxidation performance and NH_3_-SCR performance, thus enabling NH_3_-SCO and NH_3_-SCR reactions to achieve highly efficient synergy. In addition, the V_0.5_/Pt/TiO_2_ catalyst had a relatively high specific surface area, abundant surface acid sites, good redox properties, and reaction pathways that are more favorable for the generation of N_2_, which are also the reasons for its excellent N_2_ selectivity.

## Data Availability

The original contributions presented in this study are included in the article and Supplementary Materials. Further inquiries can be directed to the corresponding author.

## Supplementary Materials

The following supporting information can be downloaded at https://www.mdpi.com/article/10.3390/ma18081782/s1, Figure S1: (a) NH_3_ conversion efficiencies and (b) N_2_ selectivities of V_0.5_/Pt/TiO_2_ catalyst in the repeatability test.; Table S1: NH_3_-SCO performance comparison for V_0.5_/Pt/TiO_2_ catalyst and other catalysts in previous work; Table S2: Specific surface areas and average pore sizes of V*_x_*/Pt/TiO_2_ catalysts; Table S3: XPS results of V*_x_*/Pt/TiO_2_ catalysts; Table S4: H_2_ consumption values of V*_x_*/Pt/TiO_2_ catalysts. References [55,56,57,58,59,60,61,62] are cited in the Supplementary Materials.
