# Supplementary material for: Unraveling the Mechanism of High N2 Selectivity in Ammonia Selective Catalytic Oxidation on Pt-V Tandem Catalyst
Source: Materials (Basel). 2025 Apr 14;18(8):1782. doi: 10.3390/ma18081782 (PMC12028860; doi:10.3390/ma18081782)
Supplement: Supplementary file 1 [file materials-18-01782-s001.zip › materials-3559060-supplementary.pdf]

Supplementary Material

Unraveling the Mechanism of High N_2 Selectivity in Ammonia Selective Catalytic Oxidation on Pt-V Tandem Catalyst

Yu Gao [1,*], Pingshang Li [2] and Wan Mei [2]

1 China Waterborne Transport Research Institute, Beijing 100083, China

2 School of Environment, Beijing Jiaotong University, Beijing 100044, China

Corresponding author:

* Correspondence: gaoyu@wti.ac.cn

Contents

Section S1. Repeatability tests results 3

Section S2. Catalysts preparation 3

Section S3. Catalyst characterization...5

Section S4. NH_3-SCO performance comparison 8

Section S5. Specific surface area and average pore size 10

Section S6. XPS data 10

References...12

Section S1. Repeatability tests results

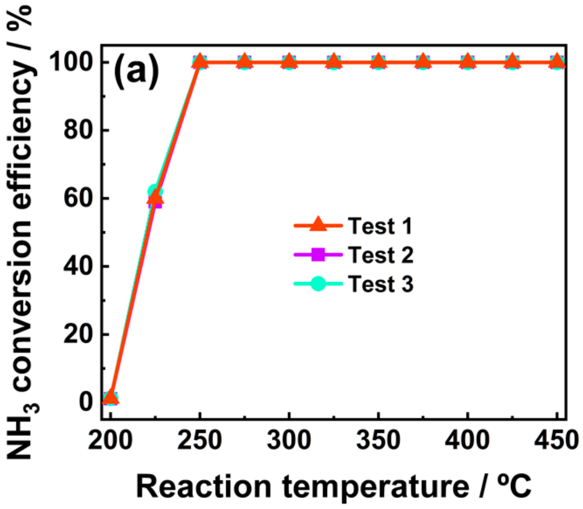

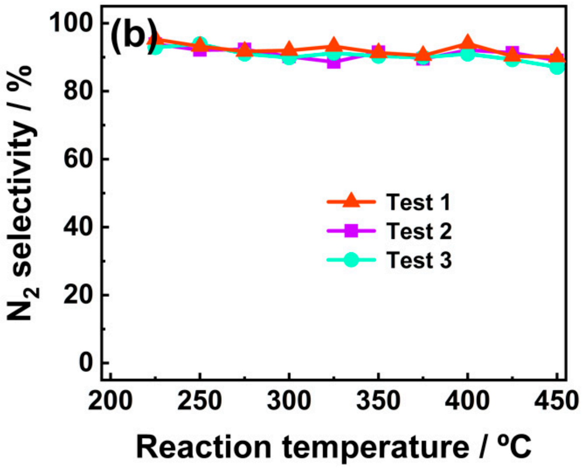

Figure S1. (**a**) NH_3 conversion efficiencies and (**b**) N_2 selectivities of $V_{0.5}$/Pt/TiO_2 catalyst in the repeatability test.

Section S2. Catalysts preparation

The preparation conditions of catalysts in this work referred to our previously published article (DOI: 10.1039/D3NJ01886E).

URL of main text:

https://doi.org/10.1039/D3NJ01886E

URL of corresponding Supporting Information:

https://www.rsc.org/suppdata/d3/nj/d3nj01886e/d3nj01886e1.pdf

The detailed preparation processes of the catalysts in this work are as follows:

- Pt/TiO_2 catalyst

Pt/TiO_2 catalyst with 0.04 wt.% Pt was synthesized by using the wet impregnation method. A total of 0.0044 g of $Pt(NO_3)_2$ solution (AR, 18.02 wt. % Pt, Aladdin Reagent Co.Ltd.) was diluted in 10 ml deionized water (15 MΩ·cm). Then, 2 g TiO_2 powder (P25, Degussa, contains 79 wt.% anatase and 21 wt.% rutile) was added to diluted $Pt(NO_3)_2$ solution under vigorous stirring. After stirring for 2 h at room temperature, the suspension solution was dehydrated by rotary evaporation and further dried at 120 ºC for 6 h. Finally, the obtained solid was calcined at 500 ºC for 3 h.

- V/TiO_2 catalyst

V/TiO_2 catalyst with 0.5 wt.% V was synthesized via the wet impregnation method. Firstly, 0.554 g $C_2H_2O_4•2H_2O$ (AR, 99.9 % purity, Sinopharm Chemical Reagent Co.Ltd.) and 0.026 g NH_4VO_3 (AR, 99.95 % purity, Sinopharm Chemical Reagent Co.Ltd.) were dissolved in 10 ml deionized water to prepare V precursor solution. Then, 2 g TiO_2 powder (P25, Degussa, contains 79 wt.% anatase and 21 wt.% rutile) was added to the above solution, followed by stirring vigorously for 2 h at room temperature. After that, the obtained suspension solution was initially dehydrated by rotary evaporation and further dried at 120 ºC for 6 h. Finally, the obtained solid was calcined at 500 ºC for 3 h.

- $V_x/Pt/TiO_2$ catalysts

$V_x/Pt/TiO_2$ catalysts with 0.1 wt.%, 0.3 wt.%, 0.5 wt.%, 0.7 wt.%, and 0.9 wt.% of V were prepared by impregnating the Pt/TiO_2 catalyst in V precursor solution. Firstly, 0.554 g $C_2H_2O_4•2H_2O$ (AR, 99.9 % purity, Sinopharm Chemical Reagent Co.Ltd.) and a certain amount of NH_4VO_3 (AR, 99.95 % purity, Sinopharm Chemical Reagent Co.Ltd.) were dissolved in 10 ml deionized water to prepare V precursor solution. Then, 2 g Pt/TiO_2 catalyst was added to 10 ml V precursor solution, followed by stirring vigorously for 2 h at room temperature. Subsequently, the obtained suspension solution

was dehydrated by rotary evaporation, and further dried at 120 ºC for 6 h. Finally, the obtained solid was calcined at 500 ºC for 3 h.

Section S3. Catalyst characterization

A series of characterization methods were employed to investigate the structure–efficiency relation of the $V_x/Pt/TiO_2$ catalyst. The details were as follows [1]. The textural properties of catalysts were evaluated by using a physisorption instrument (ASAP 2020 Plus, Micromeritics) at liquid nitrogen temperature (-196 ºC). The specific surface areas of catalysts were calculated by the N_2 adsorption–desorption isotherm using the Brunauer–Emmett–Teller (BET) equation. The average pore diameter, pore volume, and pore size distribution were calculated by the N_2 desorption isotherm using Barrett–Joyner–Halenda (BJH) model.

The X-ray diffraction (XRD) test of catalysts was carried out on an X-ray diffraction meter (Empyrean, PANalytical) using Cu Kα (λ=0.154 nm) as the radiation source. The XRD diffractograms of catalysts were recorded in the 2θ range of 20-80 º with a scanning interval of 0.02 º.

X-ray photoelectron spectra (XPS) of catalysts were measured by using a photoelectron spectrometer (AXIS-ULTRA DLD-600W, Shimadzu) with Al Kα as the radiation source. The binding energies of different elements were calibrated by C 1s peak at 284.6 eV. XPS tests in this work were performed with Constant Analyzer Energy (CAE) analyzer mode, and a pass energy of 30 eV was used. In addition, the energy step size was 0.1 eV, and the number of energy steps was 201. The total acquisition time was 20.1 s and the spot size was 500 μm.

Transmission electron microscopy (TEM) images of catalysts were measured on a JEM-2100F electron microscope (JEOL，Japan). The acceleration voltage was 200 kV,

and the surface chemical analysis was conducted by using an energy-dispersive X-ray spectrometer (EDX).

H_2-temperature programmed reduction (H_2-TPR), NH_3-temperature programmed desorption (NH_3-TPD), and oxygen programmed desorption (O_2-TPD) were conducted on a chemisorption analyzer (Autochem II 2920, Micromeritics).

Before each H_2-TPR test, the sample was flushed in He stream (50 mL/min) at 300 ºC for 30 min and then cooled down to 50 ºC. The reduction reaction proceeded from 50 to 800 ºC with a heating rate of 10 ºC/min. A mixture gas flow (50 mL/min) of 5 % H_2 and 95 % Ar was used as reducing gas [15].

For NH_3-TPD experiments, each sample was pretreated in He stream (50 mL/min) for 30 min at 200 ºC, and saturated with NH_3 at 50 ºC for 1 h. Then, the sample was treated by He stream at 50 ºC for 1 h to eliminate physically absorbed NH_3. Finally, the sample was heated from 50 to 500 ºC at a ramping rate of 10 ºC/min in He stream (50 mL/min) and NH_3-TPD data were recorded in the meantime.

For O_2-TPD experiments, each sample was pretreated in He stream (50 mL/min) for 30 min at 200 ºC, and saturated with O_2 at 50 ºC for 1 h. Then, the sample was treated by He stream at 50 ºC for 1 h. Finally, the sample was heated from 100 to 900 ºC at a ramping rate of 10 ºC/min in He stream (50 mL/min) and O_2-TPD data were recorded at the same time.

The in situ DRIFTS measurements were carried out on an FT-IR spectrometer (iS50, Thermo Fisher Scientific) equipped with a reaction cell with a KBr window (Praying Mantis, Harrick) and a program temperature controller. Typically, samples were pretreated in N_2 flow at 200 ºC for 30 min and then heated to 350 ºC to record the background spectra. Subsequently, in situ DRIFTS spectra were recorded after the reaction gas was introduced into the reaction cell.

The in situ DRIFTS experimental processes of reaction between NH_3 and O_2 were as follows: Firstly, catalysts were flushed by N_2 (100 mL/min) at 200 ºC. Then, catalysts were co-adsorbed with 10 % O_2 (50 mL/min) and 3000 ppm NH_3 (50 mL/min) for 30 min for saturation. Meanwhile, the IR spectra were recorded as a function of time [1]. After that, the reaction cell was purged with N_2 (100 mL/min) for 30 min to eliminate physically adsorbed O_2 and NH_3.

Section S4. NH_3-SCO performance comparison

The comparison for NH_3-SCO performance of $V_{0.5}/Pt/TiO_2$ catalyst and other catalysts in previous work was shown in Table S1. T_{100} represents the corresponding reaction temperature when NH_3 removal efficiency was 100 %.

Table S1. NH_3-SCO performance comparison for $V_{0.5}/Pt/TiO_2$ catalyst and other catalysts in previous work.

Catalyst	Synthesis process	Experimental conditions	T_{100} (ºC)	N_2 selectivity at T_{100} (%)	Ref.
$V_{0.5}/Pt/TiO_2$ (0.04 wt.% Pt)	Two-step impregnation	NH_3 : 3000 ppm<br>O_2 : 5 vol. %<br>N_2 as balance gas<br>GHSV : 60,000 h^{-1}	250	93	This work
Pt/γ-Al_2O_3 (0.46% wt.% Pt)	Impregnation	$[NH_3]$ = 500 ppm,<br>$[O_2]$ = 5 vol. %, N_2 as balance gas<br>GHSV = 66,000 h^{-1}	250	50	2
Pt/$CeZrO_x$ (1 wt.% Pt)	Impregnation	$[NH_3]$ = 200 ppm,<br>$[O_2]$ = 8 vol. %, N_2 as balance gas<br>GHSV = 100,000 h^{-1}	330	42	3
Pt/Anatase-TiO_2 (0.1 wt.% Pt)	Wet impregnation	NH_3 : 2000 ppm<br>O_2 : 8 vol. %<br>N_2 as balance gas<br>GHSV : 60,000 h^{-1}	275	48	4
Pt-ZSM-5 (2.55 wt.% Pt)	Ion-exchange	$[NH_3]$ = 1000 ppm,<br>$[O_2]$ = 4 vol. %, N_2 as balance gas<br>GHSV = 50,000 h^{-1}	200	71	5
Pt-V-W/TiO_2 (0.1 wt.% Pt)	Wet impregnation	NH_3 : 200 ppm<br>O_2 : 5 vol. %<br>N_2 as balance gas<br>GHSV : 100,000 h^{-1}	250	50	6

$Pt-W/ZrO_2$ (1.5 wt.% Pt)	Impregnation	$[NH_3]$ = 180 ppm, $[O_2]$ = 8 vol. %, N_2 as balance gas GHSV = 100,000 h^{-1}	300	58	7
$Ag/\gamma-Al_2O_3$	Wet impregnation	$[NH_3]$ = 500 ppm, $[O_2]$ = 10 vol. %, Ar as balance gas GHSV = 28,000 h^{-1}	180	83	8
$Ag/SiTiO_x$ (10 wt.% Ag)	Impregnation	$[NH_3]$ = 500 ppm, $[O_2]$ = 10 vol. %, N_2 as balance gas GHSV = 28,000 h^{-1}	200	63	9
$CuFeO_x$	Sol–gel	$[NH_3]$ = 800 ppm, $[O_2]$ = 3 vol. %, N_2 as balance gas GHSV = 90,000 h^{-1}	250	91	10
$CuO_x/\gamma-Al_2O_3$	Wet impregnation	$[NH_3]$ = 1000 ppm, $[O_2]$ = 10 vol. %, N_2 as balance gas GHSV = 50,000 h^{-1}	350	93	11
$FeTiO_x$	Sol–gel	$[NH_3]$ = 1000 ppm, $[O_2]$ = 3 vol. %, N_2 as balance gas GHSV = 200,000 h^{-1}	400	91	12
Mn_2O_3	Thermal decomposition	$[NH_3]$ = 500 ppm, $[O_2]$ = 3 vol. %, He as balance GHSV = 20,000 h^{-1}	210	60	13

Section S5. Specific surface area and average pore size

Table S2. Specific surface areas and average pore sizes of V_x/Pt/TiO_2 catalysts.

Sample	S_{BET} (m^2/g)	Pore volume (cm^3/g)	Pore diameter (nm)
$V_{0.1}$/Pt/TiO_2	53.56	0.32	23.37
$V_{0.3}$/Pt/TiO_2	55.23	0.31	23.55
$V_{0.5}$/Pt/TiO_2	56.27	0.35	23.38
$V_{0.7}$/Pt/TiO_2	55.93	0.33	23.21
$V_{0.9}$/Pt/TiO_2	55.88	0.33	23.18

Section S6. XPS data

Table S3. XPS results of V_x/Pt/TiO_2 catalysts.

Sample	V^{5+}/V^{3+}+V^{4+}+V^{5+} (%)	O_α/O_α+ O_β (%)	Pt^0/Pt^0+Pt^{2+}+Pt^{4+} (%)
$V_{0.1}$/Pt/TiO_2	28.20	12.82	35.56
$V_{0.3}$/Pt/TiO_2	30.52	13.22	36.03
$V_{0.5}$/Pt/TiO_2	32.65	15.23	37.66
$V_{0.7}$/Pt/TiO_2	29.83	15.09	37.02
$V_{0.9}$/Pt/TiO_2	25.22	14.62	35.23

Section S7. H_2-TPR data

Table S4. H_2 consumption values of V_x/Pt/TiO_2 catalysts

Sample	H_2 consumption value (mmol/g)
$V_{0.1}$/Pt/TiO_2	0.71
$V_{0.3}$/Pt/TiO_2	0.70
$V_{0.5}$/Pt/TiO_2	0.75
$V_{0.7}$/Pt/TiO_2	0.70
$V_{0.9}$/Pt/TiO_2	0.69

References

[1] Gao, Y.; Han, Z.; Lu, S.; Pan, X. Influence of deposition order of dual active components on the NH_3-SCO performance of the bimetallic Pt-V system supported on TiO_2. *New Journal of Chemistry* **2023**, 47, 11143-11155.

[2] Shrestha, S.; Harold, M.P.; Kamasamudram, K.; Yezerets, A. Selective oxidation of ammonia on mixed and dual-layer Fe-ZSM-5+Pt/Al_2O_3 monolithic catalysts. *Catal. Today* **2014**, 231, 105-115, https://doi.org/10.1016/j.cattod.2014.01.024.

[3] Sun, M.; Liu, J.; Song, C.; Ogata, Y.; Rao, H.; Zhao, X.; Xu, H.; Chen, Y. Different reaction mechanisms of ammonia oxidation reaction on Pt/Al_2O_3 and Pt/$CeZrO_2$ with various Pt states. *ACS Appl. Mater. Inter.* **2019**, 11, 23102-23111, https://doi.org/10.1021/acsami.9b02128.

[4] Kim, G.J.; Kwon, D.W.; Shin, J.H.; Kim, K.W.; Hong, S.C. Influence of the addition of vanadium to Pt/TiO_2 catalyst on the selective catalytic oxidation of NH_3 to N_2. *Environ. Technol.* **2019**, 40, 2588-2600, https://doi.org/10.1080/09593330.2018.1554004.

[5] Sun, M.; Wang, S.; Li, Y.; Wang, Q.; Xu, H.; Chen, Y. Promotion of catalytic performance by adding Cu into Pt/ZSM-5 catalyst for selective catalytic oxidation of ammonia. *J. Taiwan Inst. Chem. E.* **2017**, 78, 401-408, https://doi.org/10.1016/j.jtice.2017.06.045.

[6] Byun, S.W.; Lee, S.J.; Kim, M.; Bae, W.B.; Shin, H.; Hazlett, M.J.; Kang, D.; Tesfaye, B.; Park, P.W.; Kang, S.B. High N_2 selectivity of Pt-VW/TiO_2 oxidation catalyst for simultaneous control of NH_3 and CO emissions. *Chem. Eng. J.* 2022, 444, 136517, https://doi.org/10.1016/j.cej.2022.136517.

[7] Sun, M.; Wang, S.; Li, Y.; Xu, H.; Chen, Y. Promotion of catalytic performance by adding W into Pt/ZrO_2 catalyst for selective catalytic oxidation of ammonia. *Appl. Surf. Sci.* **2017**, 402, 323-329, https://doi.org/10.1016/j.apsusc.2016.12.241.

[8] Wang, F.; He, G.; Zhang, B.; Chen, M.; Chen, X.; Zhang, C.; He, H. Insights into the activation effect of H_2 pretreatment on Ag/Al_2O_3 catalyst for the selective oxidation of ammonia. *ACS Catal.* **2019**, 9, 1437-1445, https://doi.org/10.1021/acscatal.8b03744.

[9] Wang, F.; Ma, J.; He, G.; Chen, M.; Wang, S.; Zhang, C.; He, H. Synergistic effect of TiO_2-SiO_2 in Ag/Si-Ti catalyst for the selective catalytic oxidation of ammonia. *Ind. Eng. Chem. Res.* **2018**, 57, 11903-11910, https://doi.org/10.1021/acs.iecr.8b02205.

[10] Zhang, Q.; Wang, H.; Ning, P.; Song, Z.; Liu, X.; Duan, Y. In situ DRIFTS studies on CuO-Fe_2O_3 catalysts for low temperature selective catalytic oxidation of ammonia to nitrogen. *Appl. Surf. Sci.* **2017**, 419, 733-743, https://doi.org/10.1016/j.apsusc.2017.05.056.

[11] Gang, L.; Van Grondelle, J.; Anderson, B.G.; Van Santen, R.A. Selective low temperature NH_3 oxidation to N_2 on copper-based catalysts. *J. Catal.* **1999**, 186, 100-109, https://doi.org/10.1006/jcat.1999.2524.

[12] Long, R.Q.; Yang, R.T. Selective catalytic oxidation of ammonia to nitrogen over Fe_2O_3-TiO_2 prepared with a sol-gel method. *J. Catal.* **2002**, 207, 158-165, https://doi.org/10.1006/jcat.2002.3545.

[13] Lee, J.Y.; Kim, S.B.; Hong, S.C. Characterization and reactivity of natural manganese ore catalysts in the selective catalytic oxidation of ammonia to nitrogen. *Chemosphere* **2003**, 50, 1115-1122, https://doi.org/10.1016/S0045-6535(02)00708-7.
